# Silica-induced NLRP3 inflammasome activation in vitro and in rat lungs

**DOI:** 10.1186/s12989-014-0058-0

**Published:** 2014-11-19

**Authors:** Paul M Peeters, Irene M J Eurlings, Timothy N Perkins, Emiel F Wouters, Roel P F Schins, Paul J A Borm, Wolfgang Drommer, Niki L Reynaert, Catrin Albrecht

**Affiliations:** Department of Respiratory Medicine, Maastricht University Medical Centre+ (MUMC+), Maastricht University, Maastricht, The Netherlands; IUF – Leibniz Research Institute for Environmental Medicine, Düsseldorf, Germany; Bèta Sciences and Technology, Hogeschool Zuyd, Heerlen, The Netherlands; Histo-Path GmbH, Hannover, Germany

**Keywords:** Crystalline silica, NLRP3 inflammasome, Caspase-1, IL-1β, HMGB1, bFGF, TRX, PVNO

## Abstract

**Rationale:**

Mineral particles in the lung cause inflammation and silicosis. In myeloid and bronchial epithelial cells the inflammasome plays a role in responses to crystalline silica. Thioredoxin (TRX) and its inhibitory protein TRX-interacting protein link oxidative stress with inflammasome activation. We investigated inflammasome activation by crystalline silica polymorphs and modulation by TRX *in vitro*, as well as its localization and the importance of silica surface reactivity in rats.

**Methods:**

We exposed bronchial epithelial cells and differentiated macrophages to silica polymorphs quartz and cristobalite and measured caspase-1 activity as well as the release of IL-1β, bFGF and HMGB1; including after TRX overexpression or treatment with recombinant TRX. Rats were intratracheally instilled with vehicle control, Dörentruper quartz (DQ12) or DQ12 coated with polyvinylpyridine N-oxide. At days 3, 7, 28, 90, 180 and 360 five animals per treatment group were sacrificed. Hallmarks of silicosis were assessed with Haematoxylin-eosin and Sirius Red stainings. Caspase-1 activity in the bronchoalveolar lavage and caspase-1 and IL-1β localization in lung tissue were determined using Western blot and immunohistochemistry (IHC).

**Results:**

Silica polymorphs triggered secretion of IL-1β, bFGF and HMGB1 in a surface reactivity dependent manner. Inflammasome readouts linked with caspase-1 enzymatic activity were attenuated by TRX overexpression or treatment. At day 3 and 7 increased caspase-1 activity was detected in BALF of the DQ12 group and increased levels of caspase-1 and IL-1β were observed with IHC in the DQ12 group compared to controls. DQ12 exposure revealed silicotic nodules at 180 and 360 days. Particle surface modification markedly attenuated the grade of inflammation and lymphocyte influx and attenuated the level of inflammasome activation, indicating that the development of silicosis and inflammasome activation is determined by crystalline silica surface reactivity.

**Conclusion:**

Our novel data indicate the pivotal role of surface reactivity of crystalline silica to activate the inflammasome in cultures of both epithelial cells and macrophages. Inhibitory capacity of the antioxidant TRX to inflammasome activation was evidenced. DQ12 quartz exposure induced acute and chronic functional activation of the inflammasome in the heterogeneous cell populations of the lung in associated with its crystalline surface reactivity.

**Electronic supplementary material:**

The online version of this article (doi:10.1186/s12989-014-0058-0) contains supplementary material, which is available to authorized users.

## Introduction

Exposure to respirable crystalline silica dusts is very common in a multitude of industries and occupational settings. Inhalation of fine silica, including their most abundant naturally occurring polymorphs quartz and cristobalite, can lead to progressive pulmonary fibrosis and silicosis which continues to be a problem in developed and in third world countries [1]. The presence and severity of the disease depends on many factors such as individual susceptibility, and the level and duration of exposure [2]. Pathophysiologically, many molecular and micro-environmental reactions lead to sustained inflammation, prolonged tissue damage and remodeling, which over time often lead to the development of progressive interstitial fibrosis and silica-induced lung cancer [3,4].

In recent years the role of the inflammasome pathway in silica-induced lung inflammation and tissue remodeling has been emerging [5-7]. The nucleotide-binding oligomerization domain receptor, in short NOD-like receptor (NLR), pyrin domain-containing 3 (NLRP3) inflammasome belongs to the NLR subfamily of pattern-recognition receptors [8]. This multiprotein complex consists of the NLRP3 protein itself, the adapter molecule Apoptosis-associated Speck-like protein containing a CARD (ASC) and caspase-1. Assembly of these proteins leads to conversion of the pro-inflammatory cytokines IL-1β and IL-18 into their active forms by caspase-1 and release of growth factors and alarmins including basic fibroblast growth factor (bFGF) and High mobility group protein B1 (HMGB1) into the extracellular space [9-11]. In murine models of silicosis ASC-, NLRP3- and IL-1β-deficient mice are more resistant to the infiltration of inflammatory cells, the formation of granulomas, excess collagen deposition and the development of fibrosis in response to silica instillation [6]. Moreover, it has been demonstrated that treatment with an IL-1 receptor antagonist is effective in reducing established silica-induced pulmonary fibrosis and that neutralization of IL-1β could attenuate silica-induced lung inflammation and fibrosis in mice [12,13]. These data indicate that the activation of the NLRP3 inflammasome by silica is key in the development of silicosis in mice. These studies did not however examine inflammasome activation in the time course of silicosis development, nor the cell types in which it occurred.

I*n vitro*, crystalline silica-induced inflammasome activation has been investigated profoundly in effector cells of the immune system; for example macrophages treated with silica have been shown to secrete IL-1β and IL-18 in an NLRP3-dependent manner [5-7]. Furthermore, we previously demonstrated induction of inflammasome signaling in lung epithelial cells *in vitro* in response to exposure to cristobalite silica in association with inflammatory and pro-fibrogenic mediator release in the epithelial microenvironment [14]. The exact mechanism(s) by which silica particles trigger the assembly of the inflammasome complex still remains to be elucidated. One of the proposed mechanisms links lysosomal rupture and increased intracellular reactive oxygen species (ROS) levels to the assembly of the NLRP3 multi protein complex [7,15]. Other studies have shown an association between mitochondrial ROS production and regulation of the TXNIP/thioredoxin (TRX) axis to inflammasome activation [5,16,17].

The particle surface reactivity of crystalline silica has been long recognized as an important feature to their pathogenic hazard as reviewed by several authors [18,19]. Effects of silica quartz on human lung epithelial cells and rat lung epithelial cells and macrophages have been shown to be determined by its surface reactivity. Exemplary, surface modification using various compounds including aluminium or the polymer polyvinylpyridine N-oxide (PVNO) drastically affected their ability to generate ROS and the downstream activation of pro-inflammatory signaling, as well as the development of pulmonary inflammation, toxicity and fibrosis *in vitro* and *in vivo* [20-25]*.*

The aim of this study was to comparatively investigate inflammasome activation by cristobalite silica and native quartz at equal surface area metrics in different target cells *in vitro* and to determine if surface reactivity is pivotal to this activation. In conjunction, it was investigated whether inflammasome activation could be prevented by enhancing cellular TRX levels. Secondly, although the importance of inflammasome activation to the initiation of inflammation and development of fibrosis has been shown in mice, it has not been elucidated in which cellular compartments activation occurs, whether it persists, and if surface reactivity of the particles is necessary. Therefore inflammasome activation was investigated in lungs of rats at multiple time points after exposure to quartz and the influence of the particle surface properties to this response was assessed by comparative evaluation of effects after PVNO polymer coated quartz instillation.

## Methods

### In vitro experiments

#### BEAS-2B cell culture

The non-tumorigenic Ad12-SV40 immortalized human bronchial epithelial cell line BEAS 2B (ATCC, Manassas, VA) was grown and maintained in Dulbecco’s Minimal Essential Medium (DMEM)/F12 containing 10% Fetal Bovine Serum (FBS) (CellGro® Mediatech inc, Manassas, VA), with penicillin (50 U/ml), streptomycin (100 μg/ml) (Invitrogen, Carlsbad, CA), hydrocortisone (100 μg/ml), insulin (2.5 μg/ml), transferrin (2.5 μg/ml) and selenium (2.5 μg/ml) (Sigma, St. Louis, MO). Culture flasks and plates (BD, Franklin Lakes, NJ) were pre-coated with a mixture of fibronectin (Sigma, St. Louis, MO) (0.01 mg/ml), bovine collagen type I (0.03 mg/ml) (Invitrogen, Carlsbad, CA) and bovine serum albumin (BSA, 0.01 mg/ml, Sigma, St. Louis, MO) in DMEM/F12 media for 24 h at 37°C. Prior to exposures, medium was replaced with medium containing 0.5% FBS for 4 hours.

#### THP-1 cell culture

The macrophage like cell line THP-1(ATCC) was grown in RPMI 1640 medium containing 10% FBS with penicillin (50 U/ml), streptomycin (100 μg/ml) and 2 mM L-glutamine at 37°C. Ten ng/mL phorbol myristate acetate (PMA) was used to differentiate THP-1 cells for 72 h prior to experiments.

#### Surface modification of quartz particles and exposures

To modify the surface properties, DQ12 quartz (Batch 6, IUF, Düsseldorf) particles were treated with PVNO (DQ12-PVNO) as described previous [24]. Briefly, quartz was suspended at a concentration of 5 mg/mL in 1% solutions of PVNO in distilled water, subsequently sonicated for 5 min, and agitated for 3 h at room temperature. Quartz suspended in distilled water, sonicated and agitated for the same time intervals, was used as a control. The samples were then dried and stored in aliquots in the dark until use. The surface modification procedure did not affect the size distributions of the quartz sample as confirmed by electron microscopy analysis (shown in Albrecht et al. AJRCMB [21]). The *in vitro* experiments were performed with Cristobalite silica (C & E Mineral Corp., King of Prussia, PA), the sham-coated DQ12 quartz and the PVNO coated DQ12 (DQ12-PVNO). All particles for *in vitro* experiments were UV-irradiated over night to inactivate possible contaminating endotoxin. Immediately before treatment of the cell cultures, silica particle suspensions (1 mg/mL) were sonicated for 15 min, aspirated 5 times through a 23 gauge needle and added to cell cultures. Concentrations are shown in the figures as μm^2^ particle surface area per cm^2^ cell surface area on the basis of the BET surface area.

#### Assessment of cell viability

After 24 h of exposure, cells were collected by trypsinisation (Invitrogen, Carlsbad, CA) and non-viable cells were stained using trypan blue (MP Biomedicals, Solon, OH). The proportions of stained versus unstained viable cells were determined using a hemocytometer as described previously [26].

#### TRX overexpression and recombinant protein treatment in BEAS-2B and THP-1 cells

In BEAS-2B cells, transfections were performed using Extreme gene (Roche) and 1 μg DNA 24 h prior to stimulations. Differentiated THP-1 cells were transfected with 1 μg DNA using Lipofectamine 2000 (Invitrogen). Flag-tagged human thioredoxin overexpression plasmid was a kind gift of Dr. Haendeler, Heinrich-Heine University, Dusseldorf, Germany [27]. Recombinant human TRX protein was bought from Abfrontier (Korea).

#### Caspase-1 activity assay

Caspase-1 activity was measured using a commercially available assay (R&D) following the manufacturer’s protocol.

#### ELISA

Media samples were concentrated via aceton precipition with 2 volumes of ice cold acetone per volume of medium followed by 30 minutes at −20°C and centrifugation for 10 minutes at 13.000 G. The obtained pellet was resuspended in 200 μl dilution buffer. The levels of IL-1β (Biolegend) and bFGF (Biolegend) in this concentrated cell culture media were measured using commercially available ELISAs. HMGB1 was determined using a direct ELISA protocol. In brief, after a blocking step using 5% BSA for 1 hour, the primary HMGB1 antibody (ab18256 Abcam) was incubated at a 1/1000 dilution for 16 hours at 4°C. The secondary antibody was a biotin-conjugated swine anti-rabbit used at a 1/1000 dilution. Concentrations of IL-1β, bFGF and HMGB1 were established via extrapolation from a standard curve of the appropriate recombinant protein.

#### BALF concentration

500 μL of BALF was concentrated by adding 500 μL of methanol and 125 μL of Chloroform followed by vortexing and 10 minutes centrifugation at 20,000xg. Next, the upper phase was removed and another 500 μL of methanol was added to the sample followed by vortexing and 5 min centrifugation at 20,000xg. The supernatants were discarded and the pellet was dried for 15 min at 55°C after which it was resuspended in 4X sample buffer and boiled for 5 min at 95°C for subsequent Western blotting.

#### Western blot

BEAS-2B cells were lysed in a buffer containing 20 mM Tris, 150 mM NaCl, 1% [vol/vol] Nonidet P-40, 1 mM DTT, 1% [vol/vol] Protease Inhibitor Cocktail, 1% [vol/vol] Phosphatase Inhibitor Cocktail. Total protein content was determined by the Bio-Rad DC Protein Assay kit (Bio-Rad, Hercules, CA), according to manufacturer’s instructions. 20 μg protein for WCL or 15 μL of concentrated lavage or cell culture supernatants was loaded onto polyacrylamide gels. After transfer of proteins to a nitrocellulose membrane, primary antibodies against thioredoxin (ab16965, Abcam -kindly gifted by Dr. Haendeler) and caspase-1 (sc-56036, Santa Cruz) were applied at a dilution of 1:1500 and 1:300 respectively, followed by HRP conjugated secondary antibodies. SuperSignal west femto maximum sensitivity ECL Substrate was used to visualize the proteins of interest (Thermo Scientific) and images were taken on the AIDA image analyzer.

### *In vivo* experiments

#### Animals

Female Wistar rats (8 weeks old, Janvier, Le Genest St Isle, France) were used for this study. The animals were housed and maintained in an accredited on-site testing facility, according to the guidelines of the Society for Laboratory Animals Science (GV-SOLAS). All animals were allowed food and water ad libitum. The animals were housed on hardwood bedding in plastic cages in an air-conditioned animal room (23 ± 2°C) with a regular 12 h light/dark cycle. The ethical approval for the animal study application was given by the North Rhine-Westphalia State Agency for Nature, Environment and Consumer Protection (NRW - LANUV, 23.05-230-3-27/00). The intratracheal instillation procedures were performed after anesthetization of the animals using Isofluran (Essex Pharma GmbH, Munich, Germany). For to the instillation, aliquots of DQ12 or DQ12-PVNO were resuspended in PBS at a concentration of 5 mg/ml. The suspensions were sonicated (5 min) and stirred until instillation by applying a total dose of 2 mg in 0.4 ml PBS. The applied silica dose is equivalent to about 0.5 μg/cm^2^ epithelium, considering a total alveolar surface area of a rat lung of 4000 cm^2^. Control animals received 0.4 ml PBS which was sonicated and stirred in the absence of particles.

#### Animal treatment and histopathology

Rats were sacrificed in deep anesthetisation by pentobarbital at day 3, 7, 28, 90, 180 and 360 after one single intratracheal instillation of PBS, DQ12 (2 mg) or DQ12-PVNO (2 mg) as previously published [21]. The lungs of five animals per treatment group were lavaged as described in detail elsewhere from independent rats that were not included for histopathology analysis [21,28]. The lavage fluid was spun at 500xg for 10 min (4°C) and cells were collected for enumeration of total and differential cell counts; the total recovery of the BALF was 90–95% of the instilled PBS volum. Supernatants were spun again (900 xg, 10 min,4°C) and investigated for protein and inflammatory parameters. Caspase-1 cleavage products were measured in concentrated BALF. Lung fixation was performed in 5 independent animals per treatment group, for all treatment time intervals, by airway in situ perfusion of 4% Paraformaldehyde/PBS pH 7.4 at 20 cm H_2_O pressure. Lungs were embedded in paraffin and 5 μm sections were cut for histopathological evaluation as well as immunohistochemistry.

Two sections of two lobes per animal – the left as well as the right cranial – were stained with hematoxylin & eosin (H&E) as well as Sirius red to investigate histopathological alterations. On H&E stained slides the following alterations were evaluated according to severity and distribution, based on the approach used by Porter et al. [29] as detailed in Additional file 1: Table S1 in the online supplement: bronchoalveolar hyperplasia, perivascular and peribronchiolar lymphocytic infiltration, alveolar histocytosis; lympho-histiocytosis, infiltration near hyperplasia, alveolar lipoproteinosis, cholesterol granulomas and mixed cell alveolar inflammation. Sirus red staining indicates collagen deposition and was used to score perivascular fibrosis, fibrosis in alveolar septa and granulomas.

#### Immunohistochemistry

Antigen retrieval was performed by using citrate buffer (pH 6) for 10–15 minutes at 100°C on deparaffinized and rehydrated sections. Blocking with 5% BSA/TBS for 1 h at room temperature to prevent non-specific binding was next performed. Following optimization, caspase-1 (Santa Cruz, SC-56036), IL-1β (SC-7884) and HMGB1 primary rabbit anti-rat antibodies (Abcam, ab18256) were diluted in 0.5%BSA/0.1%tween/TBS to final concentrations of 2.5 μg/ml, 5.0 μg/ml and 0.5 μg/ml respectively. After 1 hour incubations with these antibodies, sections were washed 4x with 0.1%Tw/TBS. The secondary antibody (swine anti-rabbit – biotin) was diluted 400x in 0.1%BSA/TBS and incubated for 30 minutes at room temperature. The washing step was repeated and ABC/AP (200x diluted in 0.1%Tw/TBS) was used for 30 minutes at room temperature. After washing, Vector Blue (50x diluted in 0.1 M Tris (pH = 8.2)) was added to visualize the antibody complexes. The slides are washed for 5 minutes with tap water after which nuclei were stained with Nuclear Fast Red. Finally tissue sections were treated with histosafe (3x3 minutes) and mounted with vectamount. Images are taken at 100x and 200x using Leica Qwin Pro software. An additional experiment was performed including isotype IgG control raised in the secondary antibody host and images were taken at 200x and 640x magnification using Zen software on the Axiophot Zeiss microscope. Slides were reviewed blinded and independently by two observers for assessment of immunohistochemical staining. Per section 10 fields were scored using four predetermined categories (score 0 = none, 1 = mild, 2 = moderate, 3 = severe intensity). Four different compartments were chosen to evaluate intensity of staining: ^1)^ proteinosis and intra-alveolar macrophages, ^2)^ alveolar epithelial cells, ^3)^ fibrosis in alveolar septa and ^4)^ bronchial epithelium.

A Z-Score was calculated to score the relationship of each condition (DQ12 or DQ12-PVNO exposure) to the mean in a group of scores per time point. A Z-score of 0 means the score is the same as in the control (PBS) group. Z-scores per animal were calculated by averaging caspase-1 and IL-1β expression scores for all individually assessed compartments per time point and subsequently subtracting from each value the mean, μ, of the respective PBS control value followed by dividing by the standard deviation (σ) (Z = (x-μ)/σ). Data from all animals were averaged and expressed as mean values ± SEM.

### Statistical analyses

Data were analyzed by one-way analysis of variance (ANOVA) using the Student Neuman-Keul’s test to adjust for multiple pair-wise comparisons between treatment groups, or the Student’s t- test where appropriate. The results from the histopathological scoring and IHC were evaluated using the nonparametric Mann–Whitney U-test. Differences with p-values <0.05 were considered statistically significant.

## Results

### Crystalline silica polymorph-induced inflammasome readouts are associated with caspase-1 enzymatic activity in a surface reactivity dependent manner

**Figure 1:**
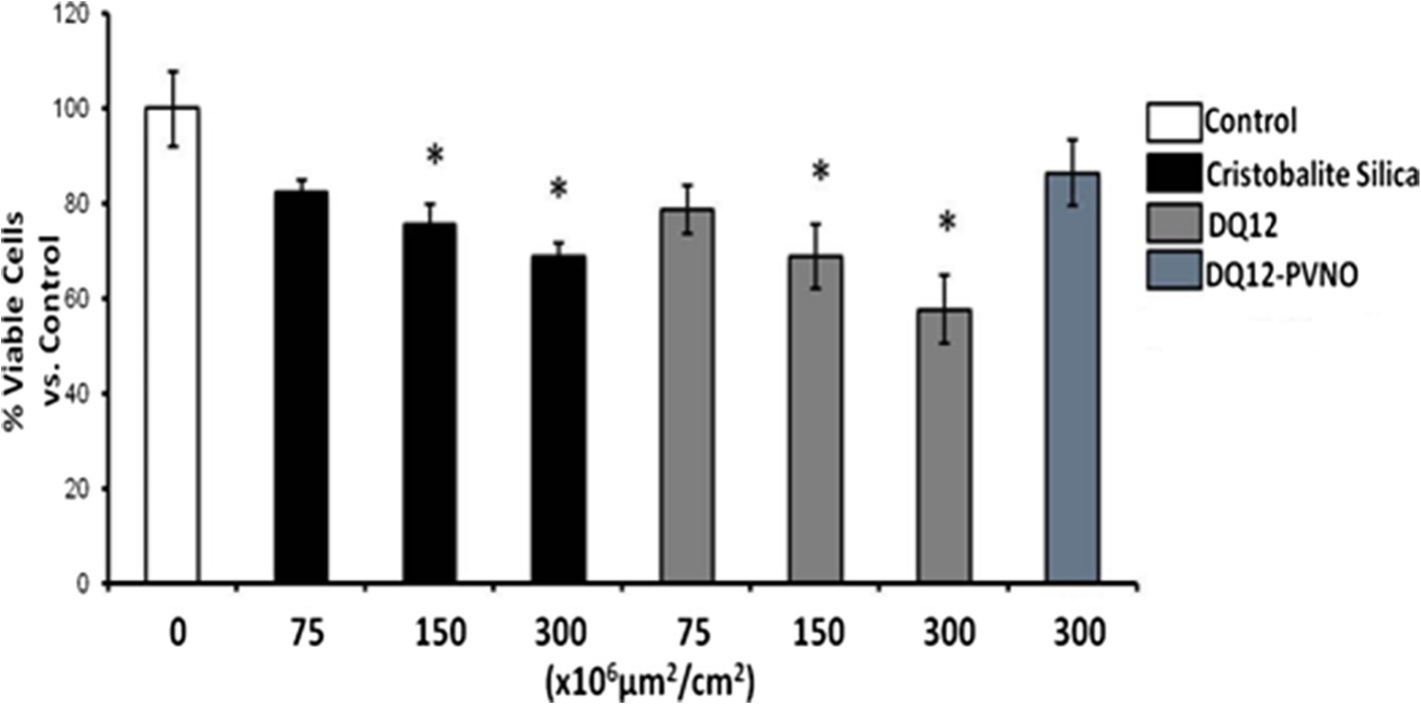
**Assessment of BEAS-2B cell viability after exposure to different silica polymorphs.** Cell viability assessed by the Trypan blue exclusion assay of cells exposed to indicated doses of cristobalite, DQ12 and DQ12-PVNO silica particles for 24 h. Results are expressed as mean percent viable cells ± SEM compared to unexposed controls (UC) and are representative of 3 independent experiments (N =3 for each treatment group in each experiment) * denotes p-value <0.05 compared to controls.

In order to compare inflammasome activation by different silica, we first determined comparative toxicity in BEAS-2B (Figure 1) and THP-1 cells (data not shown) using a range of equal surface area concentrations of DQ12 silica and cristobalite silica. Results in Figure 1 show that neither DQ12 nor cristobalite silica caused significant cell death at the lowest dose. At higher doses similar levels of cell death were observed for both polymorphs. Interestingly, PVNO-modified quartz at the highest dose did not elicit any toxicity. Comparative data were obtained in THP-1 cells. For further experiments silica concentrations of 150x10^6^μm^2^/cm^2^ were used, allowing us to investigate the inflammatory and fibrogenic effects of these mineral particles as described previously [14,30].

**Figure 2:**
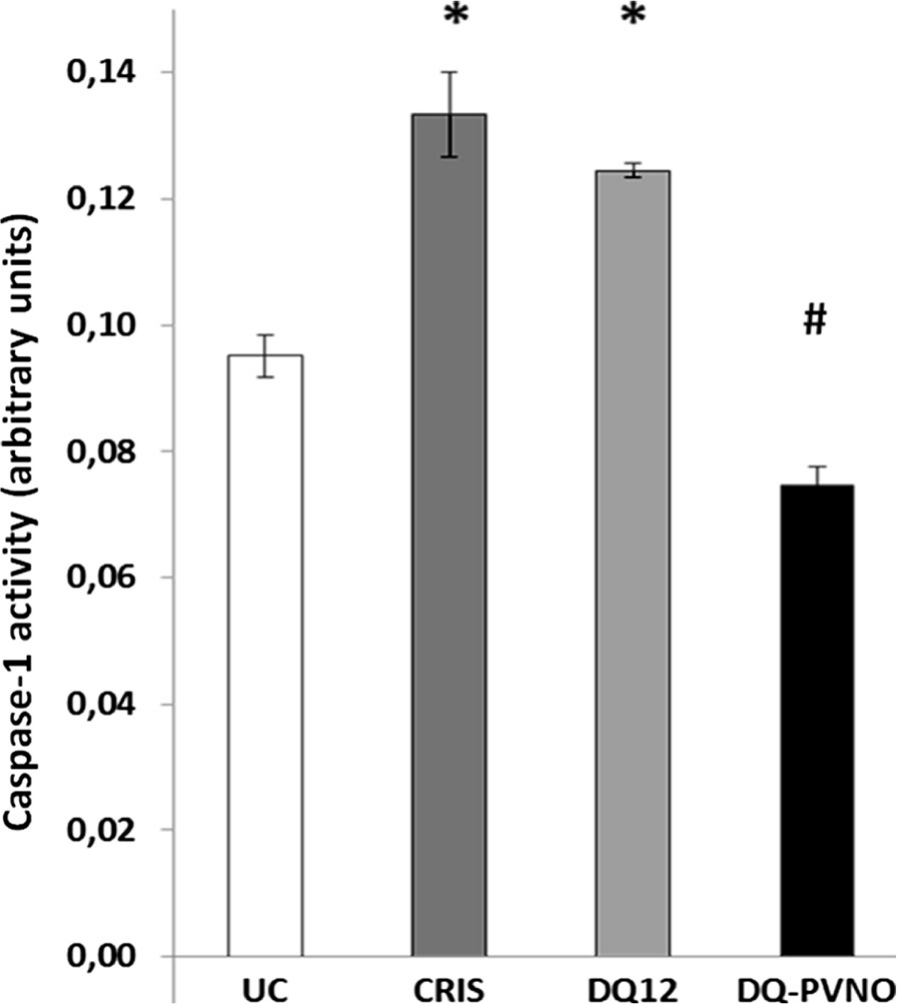
**Silica polymorph-induced caspase-1 activity in human bronchial epithelial cells.** Caspase-1 enzymatic activity was assayed in lysates of BEAS-2B cells exposed to different silica polymorphs at a dose of 150 x 10^6^ μm^2^/cm^2^ for 24 h. Caspase-1 activities are expressed as mean arbitrary units (O.D. 405 nm /total protein concentration (mg/mL)) ± SEM with * p-value <0.05 compared to UC, and # p-value <0.05 compared to DQ12.

We next assessed caspase-1 cleavage by both silica polymorphs comparatively at equal surface concentrations. Levels of the functional p20 subunit of caspase-1 in BEAS-2B indeed similarly increased after treatment with cristobalite and DQ12 for 24 h. Particle surface modification furthermore abrogated the potential of DQ12 to induce caspase-1 activation (Figure 2).

**Figure 3:**
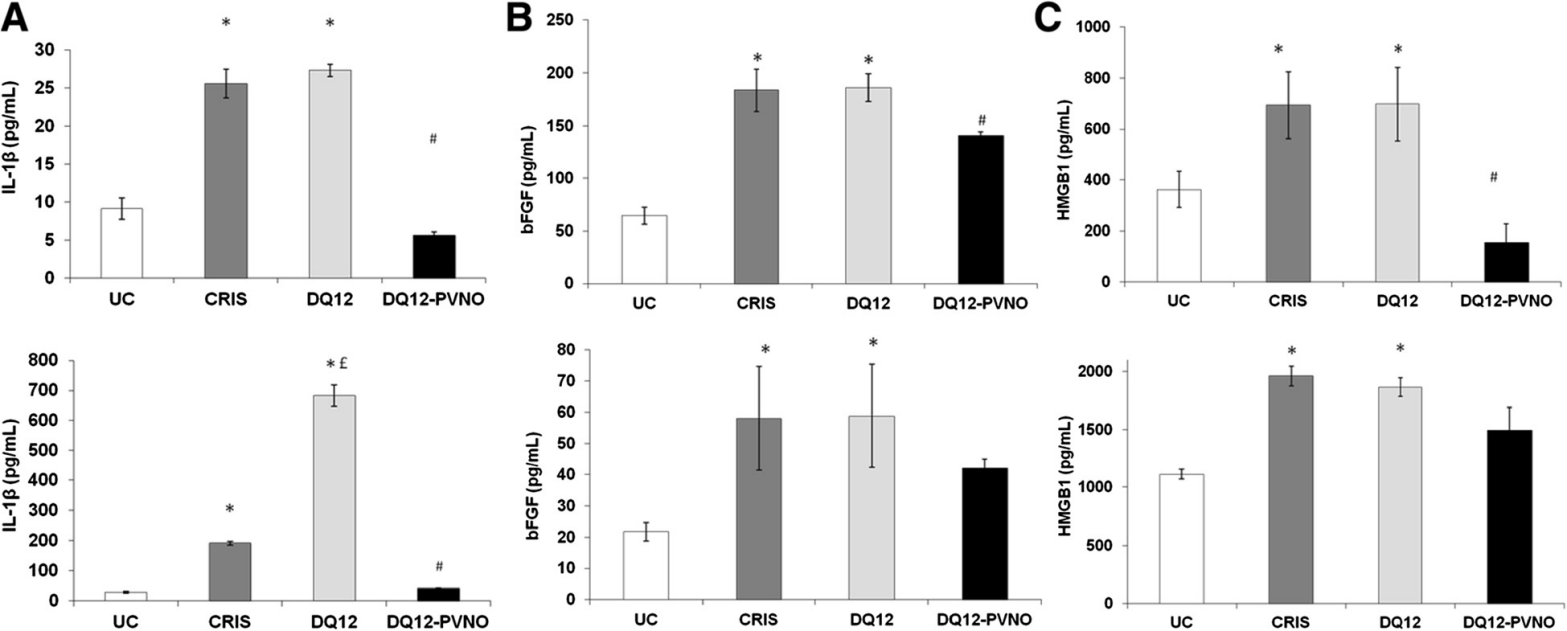
**Silica polymorphs augment release of NLRP3-associated inflammatory and fibrotic mediators as well as alarmins from lung epithelial cells and differentiated macrophages, which is dependent on surface area reactivity.** Investigation of markers for inflammasome activation in BEAS-2B (upper panels) and THP-1 (lower panels) cells following exposure to cristobalite, native and PVNO coated DQ12 silica for 24 h at a dose of 150 x 10^6^ μm^2^/cm^2^. ELISAs were performed on concentrated medium for detection of secreted IL-1β **(A)**, bFGF **(B)** and HMGB1 **(C)**. Data are presented as mean ± SEM with * p-value <0.05 compared to UC, £ p-value < 0.05 compared to SIL150 and # p-value <0.05 compared to DQ12 silica exposure.

We previously showed that release of not only IL-1β, but also of bFGF and HMGB1 by BEAS-2B and THP-1 cells in response to cristobalite silica was dependent on NLRP3. Therefore we next assessed if DQ12 similarly caused the release of these inflammasome-dependent mediators and if this then is dependent on surface reactivity as observed for caspase-1 activity in Figure 2. Indeed, results in Figure 3 demonstrate that cristobalite and DQ12 both act as inflammasome activators, causing significantly increased release of IL-1β, bFGF and HMGB1 in both cell types. Levels of these mediators induced by both polymorphs were comparable in BEAS-2B cells. In THP-1 cells, bFGF and HMGB1 levels in response to cristobalite and DQ12 were similar as well, but DQ12 was found to be a more potent inducer of IL-1β release. The differential levels of these mediators observed between the two cell types under investigation, under baseline conditions as well as in response to silica treatment are in line with our previous study [14]. Interestingly, no significant increases in mediator levels in response to DQ12-PVNO compared to untreated controls were found. Also, compared to uncoated DQ12, the response was mostly significantly dampened by surface coating in both cell types.

### Crystalline silica-induced caspase-1 enzymatic activity and inflammasome readouts are attenuated by thioredoxin

**Figure 4:**
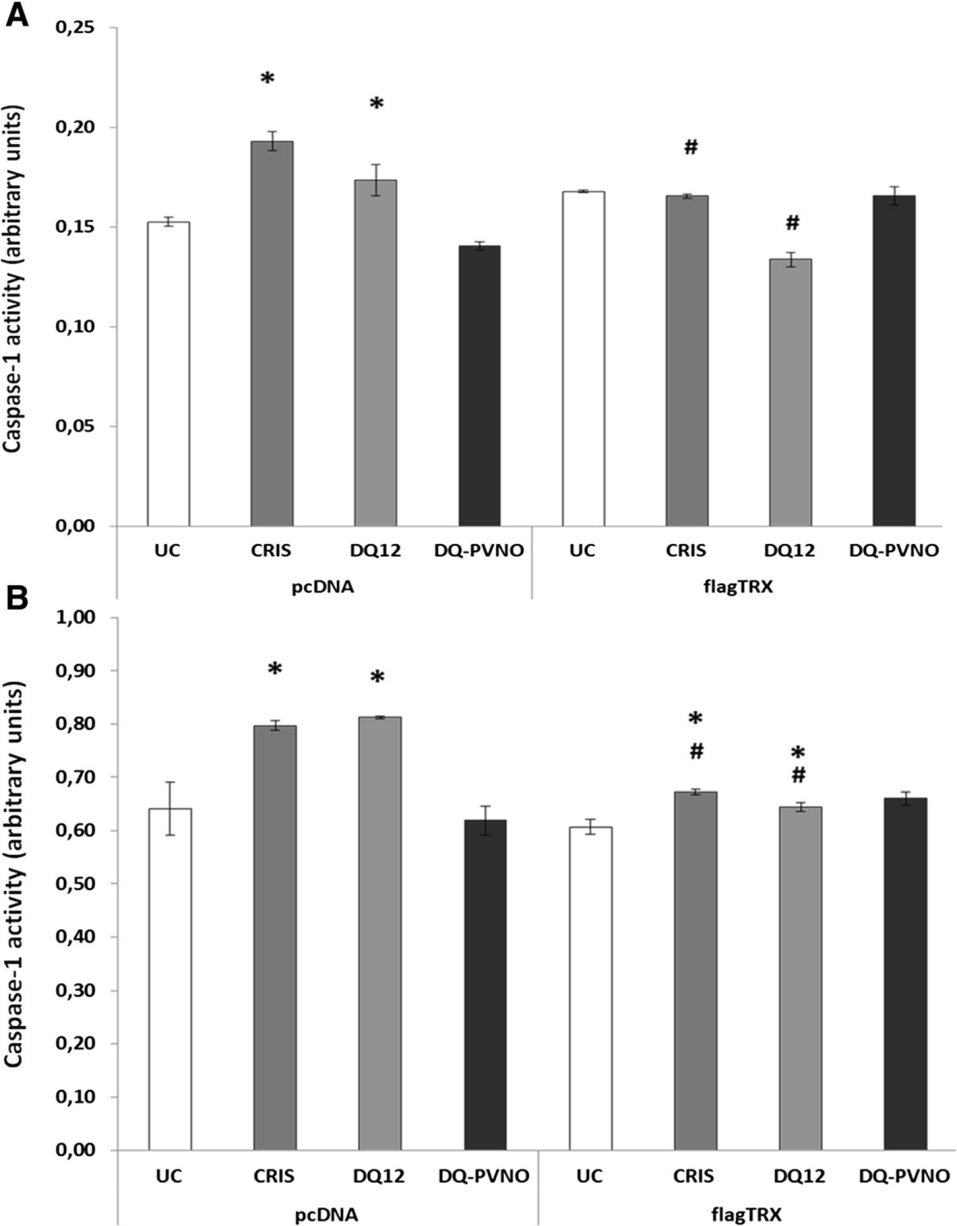
**Silica-induced caspase-1 activity in human bronchial epithelial and macrophage-like cells is attenuated by thioredoxin.** Caspase-1 enzymatic activity was assayed in cell lysates of BEAS-2B **(A)** and THP-1 cells **(B)** exposed to different silica polymorphs for 24 h at a dose of 150 x 10^6^ μm^2^/cm^2^, transiently overexpressing flag-tagged thioredoxin (flagTRX) or pcDNA empty plasmid. Caspase-1 activities are expressed as mean arbitrary units (O.D. 405 nm /total protein concentration (mg/mL)) ± SEM with * p-value <0.05 compared to appropriate controls (UC), and # p-value <0.05 compared to same treatment in pcDNA conditions.

Intrinsic and extrinsic ROS formation is thought to be an essential step in the activation of the inflammasome in response to silica. Our data, with respect to dampening of the response by surface coating, support this hypothesis. One mechanism by which ROS are linked to inflammasome activation is by oxidation mediated release of TXNIP from TRX. We thus hypothesized that increasing TRX levels could prevent inflammasome activation in response to silica treatment. The presence of TRX was measured on WCL and concentrated SN of TRX plasmid transfected and TRX protein treated BEAS-2B cells by Western blotting (Additional file 2: Figure S1). In BEAS-2B (Figure 4A) as well as in THP-1 cells (Figure 4B), silica-induced caspase-1 activity levels were significantly lower in cells overexpressing TRX compared to pcDNA controls. In addition, pretreatment of BEAS-2B cells with recombinant human TRX protein similarly attenuated caspase-1 activation in response to silica treatment (data not shown).

**Figure 5:**
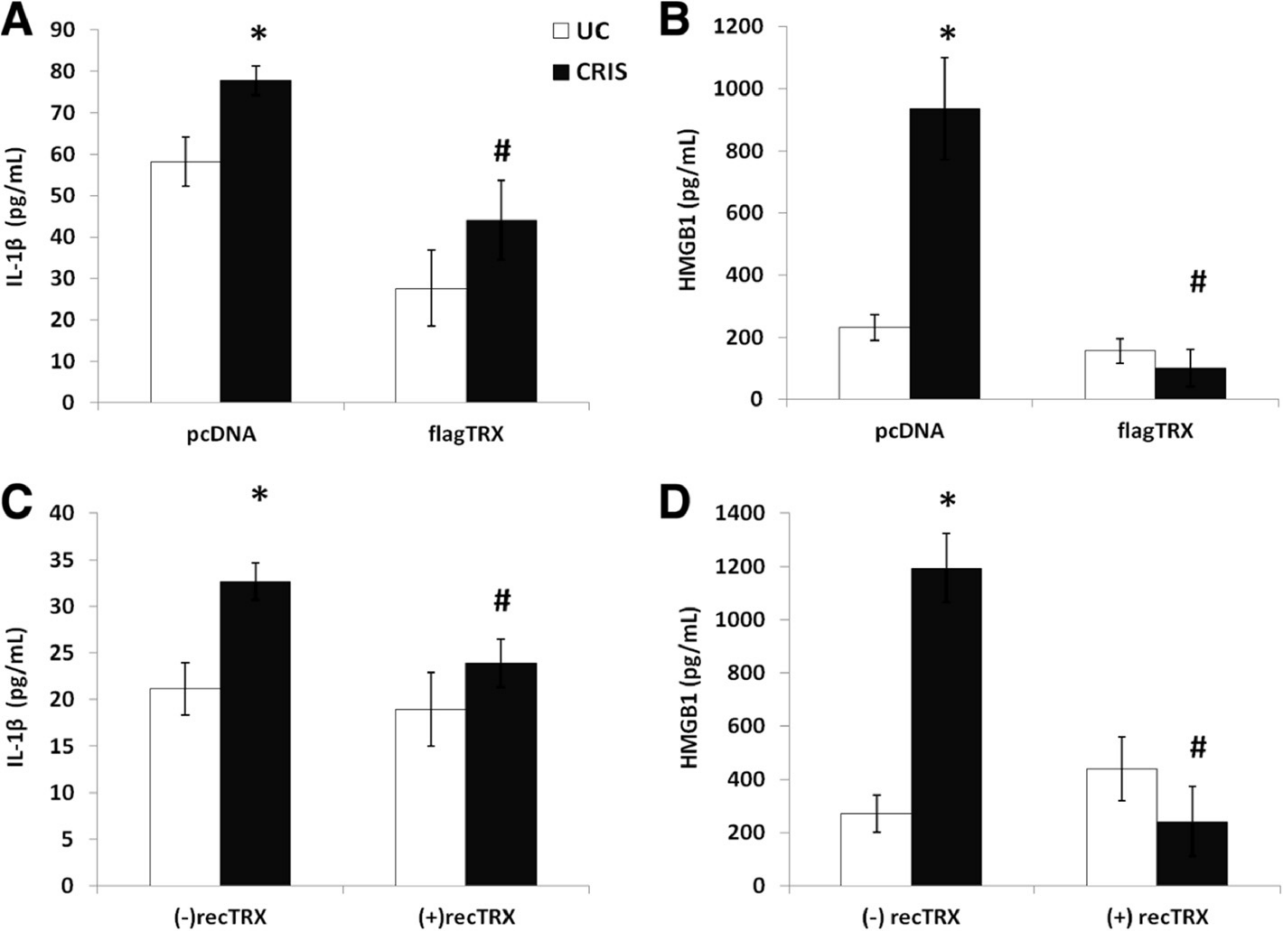
**Thioredoxin overexpression or pretreatment attenuates the release of IL-1β and HMGB1 in response to cristobalite silica.** ELISAs were performed on concentrated medium of BEAS-2B cells transiently overexpressing flagTRX (top panel) or pre-treated with 250 ng/ml recombinant human TRX (lower panel) and exposed to 150 x 10^6^ μm^2^/cm^2^ of cristobalite silica for 24 h for the detection of secreted IL-1β **(A and C)** and HMGB1 **(B and D)**. Data are presented as mean ± SEM with * p-value <0.05 compared to UC, and # p-value <0.05 compared to respective crystalline silica exposure in the pcDNA or non-TRX treated group.

To further investigate a protective role of TRX in crystalline silica-induced inflammasome signaling we determined IL-1β and HMGB1 levels in the medium of BEAS-2B cells overexpressing TRX or pretreated with recombinant human TRX protein (Figure 5). In agreement with effects observed on caspase-1 activity, both overexpression of TRX and pretreatment with recombinant TRX abrogated the release of IL-1β and HMGB1 by BEAS-2B cells following cristobalite silica exposure. We also examined bFGF release in these experiments however these growth factor levels were not significantly affected by TRX (data not shown). Present results show that by enhancing TRX levels, either genetically or by treatment with the recombinant protein it is possible to attenuate inflammasome signaling.

### Crystalline silica surface area reactivity is an important driver of lung damage, inflammation and histopathological hallmarks of silicosis *in vivo*

As particle coating has been proven to affect *in vivo* responses as well with respect to inflammation and fibrosis, we examined how different cell types in rat lung tissue are affected over time when exposed to coated and uncoated crystalline minerals.

**Figure 6:**
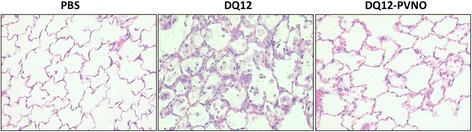
**Particle surface modification attenuates the development of silicotic tissue morphology in rat lungs.** Representative microphotographs of lung histopathology at 90 days after instillation of DQ12 or DQ12-PVNO by H&E staining. Original magnification × 200.

Representative lung tissue sections of rats exposed to PBS, coated and non-coated quartz for 90 days are shown in Figure 6. H&E staining in the DQ12 exposed group demonstrated massive inflammation and particle-laden macrophages in the alveoli. Next to influx and accumulation of lymphocytes and neutrophils in the interstitium, alveolar histiocytosis accompanied with intra-alveolar proteinosis and hyperplasia was present in the native quartz group. Figure 6 also reveals the presence of fibrosis of the alveolar septa in lungs of rats exposed to DQ12 quartz. Quartz particles coated with PVNO did not cause disintegration of the alveolar structure. Moreover, DQ12-PVNO exposure revealed a decrease in the influx of inflammatory cells and an absence of fibrogenic histopathological characteristics in our silicosis model compared to uncoated quartz. The histopathological evaluations as evaluated by H&E and Sirius Red staining and scoring thereof for all treatment times are listed in Table 1 and confirm the rapid pro-inflammatory effect of DQ12 as well as the progressive increases in pulmonary toxicity of this crystalline silica sample. By comparison, significantly less pronounced degrees of alveolar lipoproteinosis, alveolar histocytosis and bronchoalveolar hyperplasia were observed with the PVNO coated quartz with increasing time intervals. The surface modification of the DQ12 also led to lower fibrosis scores in the alveolar septa and perivascular regions.

**Table 1:** **Summary of histopathological evaluation of lung tissue of rats exposed to DQ12, DQ-PVNO or PBS**

**Alveolar lipoproteinosis**				**Mixed cell alveolar inflammation**			
	**PBS**	**DQ12**	**DQ12-PVNO**		**PBS**	**DQ12**	**DQ12-PVNO**
3d	0.0 ± 0.0	0.0 ± 0.0	0.0 ± 0.0	3d	0.0 ± 0.0	1.2 ± 0.7*	0.0 ± 0.0^#^
7d	0.0 ± 0.0	0.2 ± 0.4	0.0 ± 0.0	7d	0.0 ± 0.0	1.1 ± 0.7*	0.5 ± 0.6
28d	0.0 ± 0.0	1.0 ± 0.3**	0.3 ± 0.4^##^	28d	0.0 ± 0.0	0.8 ± 1.1	0.0 ± 0.0
90d	0.0 ± 0.0	1.3 ± 0.8*	0.0 ± 0.0^#^	90d	0.0 ± 0.0	0.5 ± 0.4	0.1 ± 0.2
180d	0.0 ± 0.0	2.0 ± 0.4**	0.3 ± 0.4^##^	180d	0.0 ± 0.0	0.0 ± 0.0	0.0 ± 0.0
360d	0.0 ± 0.0	2.6 ± 1.5**	2.9 ± 1.5^$$^	360d	0.0 ± 0.0	0.6 ± 1.0	0.0 ± 0.0
**Alveolar histocytosis**				**Brochoalveolar hyperplasia**			
	**PBS**	**DQ12**	**DQ12-PVNO**		**PBS**	**DQ12**	**DQ12-PVNO**
3d	0.0 ± 0.0	0.8 ± 0.7	0.1 ± 0.3	3d	0.0 ± 0.0	0.7 ± 0.9	0.3 ± 0.7
7d	0.3 ± 0.7	1.5 ± 0.7	0.5 ± 0.4^#^	7d	0.9 ± 1.8	2.2 ± 1.4	0.6 ± 0.8
28d	1.0 ± 0.4	3.4 ± 1.4*	1.4 ± 0.8^#^	28d	1.0 ± 0.4	3.1 ± 1.8	1.4 ± 1.2
90d	0.4 ± 0.7	3.8 ± 0.6**	1.0 ± 0.7^##^	90d	0.8 ± 1.0	2.6 ± 1.4	0.6 ± 0.3
180d	0.8 ± 0.4	2.4 ± 0.6*	*2.5 ± 1.0* ^*$*^	180d	0.1 ± 0.3	2.3 ± 1.0**	*2.3 ± 0.7* ^*$$*^
360d	0.3 ± 0.3	3.0 ± 0.6**	*2.0 ± 0.4* ^*$$,#*^	360d	0.0 ± 0.0	5.5 ± 1.1**	*3.9 ± 1.0* ^*$$,#*^
**Fibrosis alveolar septa**				**Perivascular fibrosis**			
	**PBS**	**DQ12**	**DQ12-PVNO**		**PBS**	**DQ12**	**DQ12-PVNO**
3d	0.0 ± 0.0	0.1 ± 0.3	0.0 ± 0.0	3d	1.0 ± 0.2	1.0 ± 0.0	0.9 ± 0.2
7d	0.0 ± 0.0	0.0 ± 0.0	0.0 ± 0.0	7d	0.6 ± 0.2	1.0 ± 0.0*	1.0 ± 0.0
28d	0.0 ± 0.0	0.4 ± 0.6	0.0 ± 0.0	28d	1.1 ± 0.2	1.3 ± 0.4	1.1 ± 0.2
90d	0.0 ± 0.0	1.0 ± 0.8*	0.0 ± 0.0^#^	90d	1.0 ± 0.0	1.4 ± 0.4	1.0 ± 0.0
180d	0.2 ± 0.2	1.4 ± 1.3	0.4 ± 0.4	180d	0.8 ± 0.2	1.6 ± 0.2**	*1.5 ± 0.4* ^*$*^
360d	0.7 ± 0.5	2.1 ± 0.2**	1.3 ± 0.5^#^	360d	1.1 ± 0.4	2.3 ± 0.2**	*1.9 ± 0.4* ^*$*^

Histopathological evaluation of 2 lung lobes (left, right cranial) each stained with H&E and Sirius red 3, 7, 28, 90, 180 and 360 days after one single intratracheal instillation of PBS, DQ12 or DQ12-PVNO. For histopathological scoring, data were calculated as the sum of lesion severity and distribution. The mean of the two lobes was calculated and data expressed as mean ± sd of 5 animals per treatment and time point. DQ12 vs. PBS: * p <0.05; ** p <0.01; DQ12-PVNO vs. PBS: ^$^ p < 0.05, ^$$^ p > 0.01; DQ12 vs. DQ12-PVNO: # p <0.05; ## p <0.01.

### Particle surface modification attenuates inflammasome activation in the BALF and rat lung tissue

**Figure 7:**
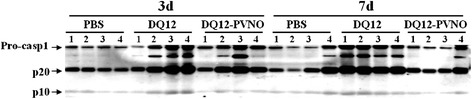
**Detection of caspase-1 cleavage products in BALF of silica exposed rats.** Cleaved caspase-1 p20 and p10 subunits were detected in concentrated lavage fluid of rats exposed to PBS, DQ12 or DQ12-PVNO for 3 and 7 days by Western blotting (4 animals per group).

The contribution of inflammasome activation to the development of silicosis has been shown in a mouse model; however caspase-1 activity and its particle surface reactivity dependency in the BALF of rats was not demonstrated yet, neither has it been localized in an animal model of silicosis. In Figure 7 we showed increased levels of caspase-1 cleaved subunits in the BALF of DQ12 exposed rats. This effect was partly abrogated under conditions where rats were treated with the polymer-modified quartz.

**Figure 8:**
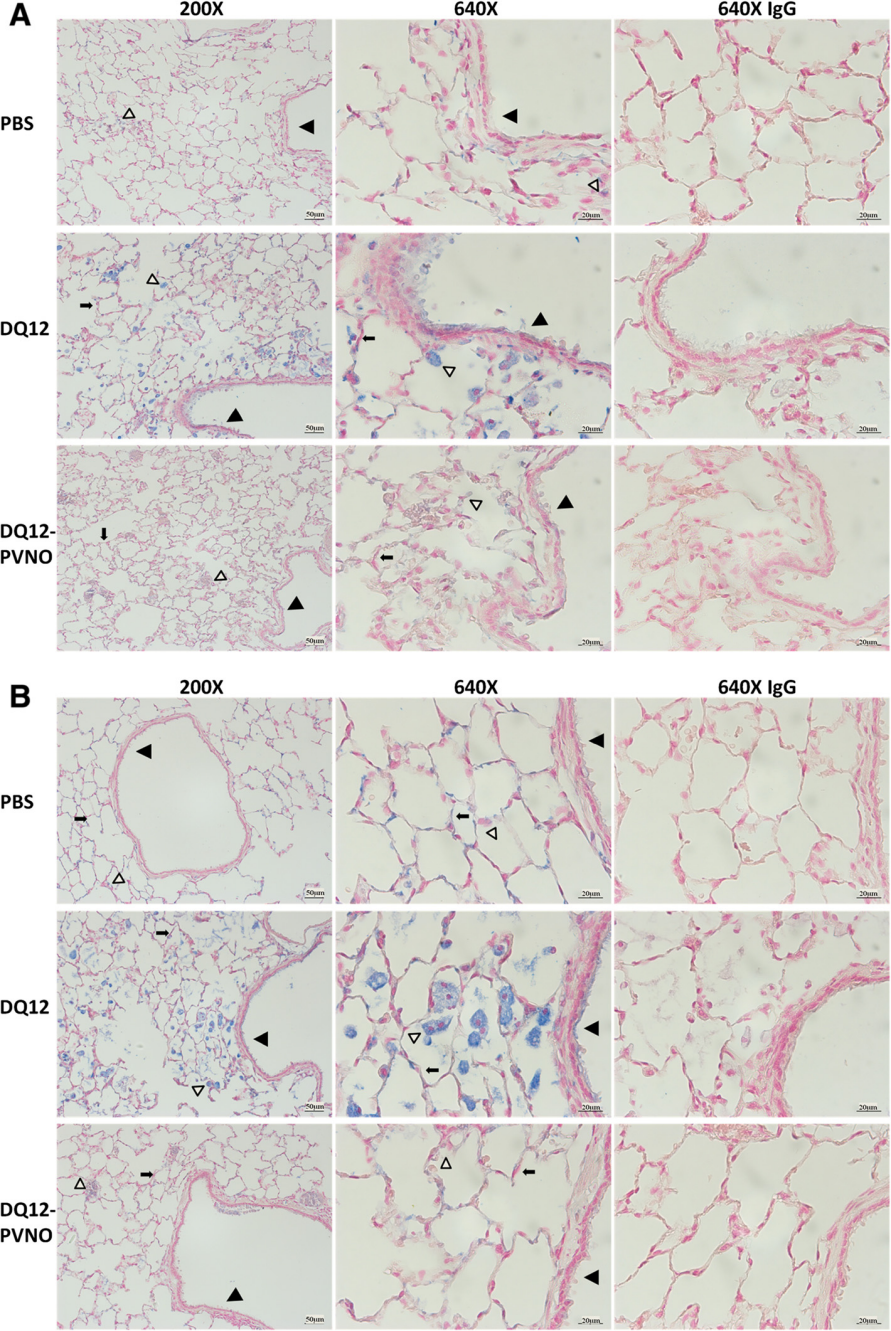
**Particle surface modification modulates inflammasome activation in rat lungs.** Immuno-histochemical staining of caspase-1 **(A)** and IL-1β **(B)** in PBS control vs. DQ12 or DQ12-PVNO treated rats at 180 days after exposure along with corresponding IgG control staining. Images are representative of 5 rats per group. Caspase-1 and IL-1β are stained in blue. Slides were counterstained using Nuclear Fast Red. Arrows indicate specific cell types, i.e. bronchial epithelium (solid triangle), alveolar epithelial cell (arrow), macrophages (open triangle).

Another goal was to localize inflammasome activation in the lung tissue of the rat model used here and secondly to examine if this activation was dependent on surface reactivity. Immunohistochemistry for caspase-1 and IL-1β as pivotal readouts of inflammasome activation, to assess and localize activation of the inflammasome complex were performed. Clearly increased expression levels of caspase-1 and IL-1β in alveolar, bronchial epithelial, myeloid and endothelial cells in lungs of rats that were exposed to DQ12 for 180 days is evident in Figure 8. Additionally, exudates in alveolar proteinosis-characterized areas were significantly stained more intensely for these cleaved subunits and pro-forms of the protease and cytokine. A significant reduction in caspase-1 and IL-1β staining was observed in lung tissue of the PVNO-coated rats. Immunohistochemical staining performed on lung tissue obtained at other time points under investigation reveal similar results (Additional file 3: Figure S2A-F).

**Figure 9:**
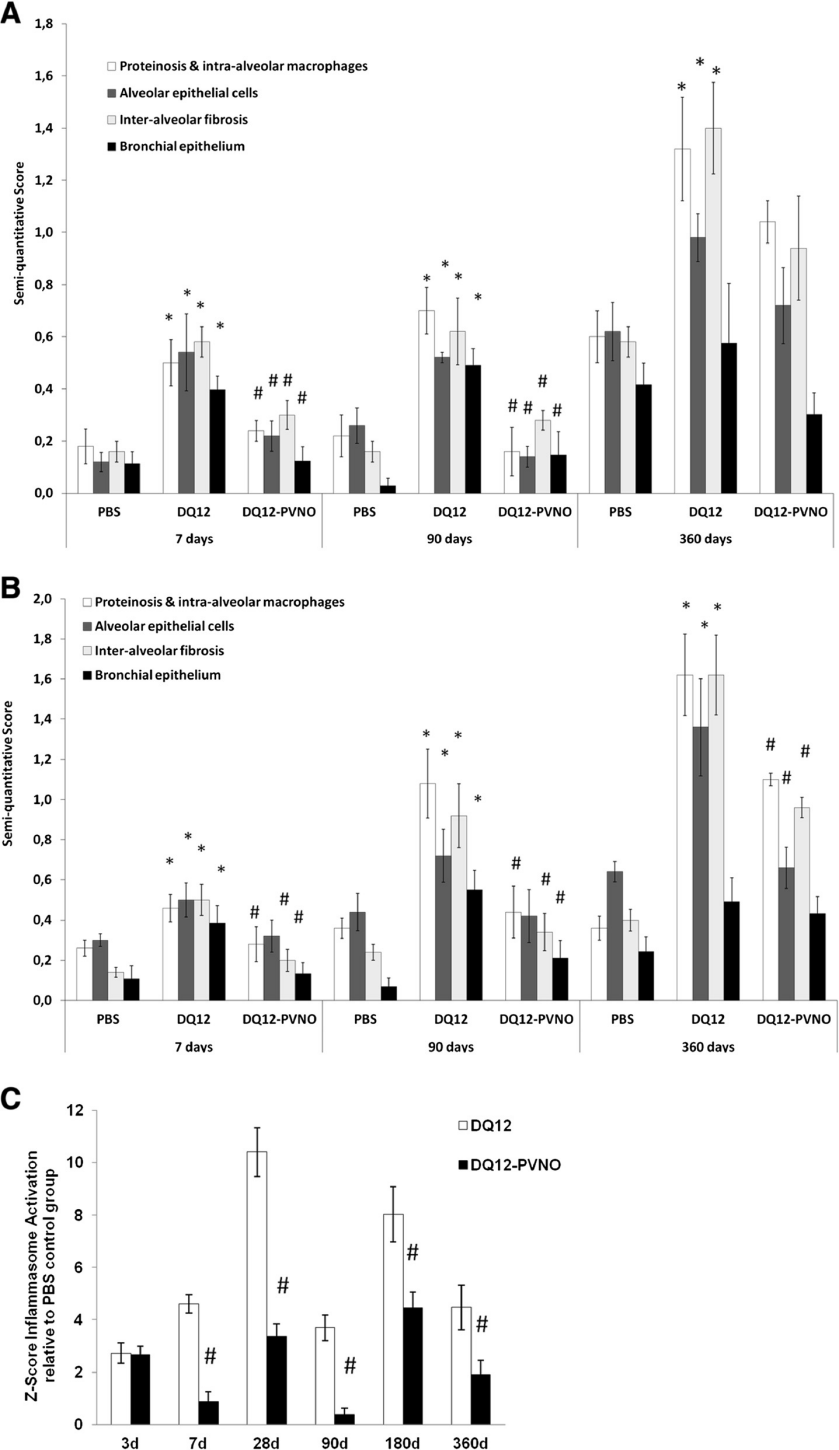
**Native quartz exposure leads to sustained inflammasome activation in different cellular and interstitial compartments of the rat lung, which is attenuated by surface modification.** Results represent semi-quantitative scoring of caspase-1 **(A)** and IL-1β **(B)** immunohistochemistry in different compartments of lungs at acute (7 days) and chronic inflammatory conditions (90 and 360 days) following a single silica or PBS challenge. Data are presented as mean ± SEM with * p-value <0.05 compared to PBS, and # p-value <0.05 compared to DQ12 exposure. **C**, Z-scores for total inflammasome (of caspase-1 and IL-1β combined) staining intensities over all compartments in DQ12 and DQ12-PVNO groups normalized to the average inflammasome staining of the PBS group at each time point with # p-value <0.05 comparing DQ12-PVNO vs UC with DQ12 vs UC.

Inflammasome activation, determined by caspase-1 and IL-1β expression levels, was semi-quantitatively scored, in different predefined compartments; ^1)^ alveolar proteinosis and intra-alveolar macrophages, ^2)^ staining in alveolar epithelial cells, ^3)^ inter-alveolar fibrosis and ^4)^ bronchial epithelium. Figure 9A demonstrates that in each of the 4 compartments caspase-1 expression levels were significantly increased in the DQ12 treated group acutely and further increased at the later time points. The expression levels of caspase-1 were much lower at any time point in the polymer coated quartz exposed group compared to the native quartz exposed group. For day 3, 28, 180 similar data were obtained (not shown). Scoring of the expression of the pro-form and cleaved IL-1β subunits in these rat lung tissue sections provided data revealing similar patterns regarding inflammasome activation by crystalline silica including time-dependent increases and blunting by surface modified quartz exposure (Figure 9B). Finally, an overall score for inflammasome activation was calculated based on these two immunohistochemical stainings individually assessed in the 4 separate compartments per time point for each quartz treatment in relation to the PBS controls (Figure 9C). A Z-score of 0 indicates that the overall inflammasome score in that specific condition does not differ from the mean score of the control group. DQ12 exposure thus induced inflammasome activation at all time points, and this level of inflammasome activation was largely attenuated in the uncoated quartz group.

## Discussion

Prolonged exposure to silica can lead to the development of silicosis, an irreversible, fibrotic pulmonary disease. Inflammasome activation by silica has been reported in myeloid and epithelial cells of the lung *in vitro* and is crucial to development of silicosis in mice [6,13]. In this study we demonstrated that the inflammasome pathway is activated in inflammatory as well as structural cells in rat lungs up to 1 year after a single intratracheal instillation of crystalline silica in association with pulmonary inflammation and fibrogenesis. Further, we found these effects to be largely absent when PVNO -polymer modified quartz was instilled, indicating the importance of the surface modification *in vivo* to both the induction of inflammasome activation as well as associated inflammatory and fibrotic responses. It is of notice that the PVNO-coated material has lower effects although, especially at later time points post instillation, some effects are emerging (180 and 360 days). This could perhaps be due to the removal of some of the coating over time. In fact the data show that PVNO-coating blunts much of the toxic responses of the DQ12, however, it does not turn the DQ12 into an “inert” dust.

Secondly, in epithelial and myeloid cells inflammasome activation was demonstrated *in vitro* to a similar extent by different silica polymorphs, which was also found to be critically dependent on surface reactivity of the crystalline dust. Additionally, inflammasome-dependent mediator release could be reduced not only by surface modification, but also by enhancing endogenous thioredoxin levels by overexpression or exogenous administration of this antioxidant.

In the *in vivo* study increased activation of caspase-1 and increased levels of IL-1β were observed in response to DQ12 treatment in different cellular compartments of the lung, at acute, as well as chronic timepoints. Here, pro- as well as cleaved/active subunits of these proteins, so the total amount of this protein is detected in the rat lung tissues. However, it has been shown that when activated, increased mRNA expression of both capase-1 and IL-1β occurs as a positive feedback mechanism to replenish protein levels and provide sufficient substrate. The increased protein levels of caspase-1 and IL-1β although not specific for activated forms, can be regarded as proof of activation of the inflammasome. These data indicate a synergistic contribution of alveolar and bronchial epithelial cells as well as myeloid cells, endothelial cells and fibroblasts to the total inflammasome activation which has been reported previously in mouse models of silicosis.

*In vitro* studies have revealed that, in response to silica and other crystals, immune cells deficient in components of the NLRP3 inflammasome are incapable of secreting the proinflammatory cytokines IL-1β and IL-18 [7,14,31]. Silica-induced inflammasome activation in non-myeloid cells is emphasized in recent publications where interestingly human keratinocytes released mature, cleaved IL-1β only upon exposure to SiO2 nanoparticles, although it has not been fully characterized to what extent nano-SiO2 is crystalline [32]. Our group furthermore demonstrated the presence and functional activation of the NLRP3 inflammasome in human lung epithelial cells in response to cristobalite silica [14]. By the studies represented in this manuscript we add to these data that inflammasome activation in bronchial epithelial cells and differentiated macrophages can be induced to a similar degree by the two most common polymorphs of crystalline silica, i.e. quartz and cristobalite, to secrete IL-1β, bFGF and HMGB1 from human lung cells *in vitro.* Accordingly, from a review of *in vivo* experimental and epidemiological studies, Mossman and Glenn recently synthesized no evidence for differences between quartz and cristobalite in being pro-inflammatory and possibly fibrogenic agents [33]. Besides silica, also asbestos fibers (hydrated silicates) can prime and activate the NLRP3 inflammasome [5,34] in for instance human mesothelial cells [35]. The importance of the NLRP3 inflammasome and HMGB1 in asbestos-induced inflammation has in addition been linked to mesothelioma [36].

The polymer PVNO has been investigated for its potential prophylactic and therapeutic use in silica and silica-containing dust induced fibrosis, albeit with limited success [37-39]. Next to addition, adsorption of PVNO onto the crystalline silica surface has been shown to blunt its reactivity via a mechanism that is considered to involve H-bonding of its NO groups with the reactive silanol groups at the quartz surface. A direct coating approach of quartz with the polymer has been used by us in various investigations to address the contribution of the surface properties in silica toxicity and pathogenesis [24,25]. First, it could be demonstrated by electron spin resonance spectroscopy that PVNO coating inhibits the intrinsic ROS properties of DQ12 quartz. Subsequently, it was shown that such modification abrogates ROS-mediated induction of oxidative DNA damage responses in lung epithelial cells *in vitro* [25,40] and rat lungs *in vivo* [24]. PVNO coating has also specifically been shown to abrogate the formation of ROS by phagocytic cells and ROS levels in lavage fluid of exposed rats [20,24]. In association with this, PVNO modification also had profound effects on the induction and persistence of pulmonary inflammation [21] as well as the cellular uptake of silica particles and their clearance from rat lung [41]. This is further evidenced by our additional proof for *in vivo* surface reactivity dependent inflammasome activation by means of caspase-1 acitivity in the BALF. Our present data in addition reveal an important role for the crystalline surface of silica polymorphs in the activation of caspase-1 and in the ability to induce secretion of IL-1β, bFGF and HMGB1 from human lung epithelial and macrophage like cells *in vitro* and in inflammasome activation *in vivo*.

Activation of the NLRP3 inflammasome by silica requires both an efflux of intracellular potassium and the generation of reactive oxygen species in myeloid cells [6]. Hornung et al. added evidence that particle uptake in peripheral blood mononuclear cells subsequently leads to lysosomal damage and rupture, and that inhibition of either phagosomal acidification or cathepsin B activity impaired NLRP3 activation. Their work indicates that the NLRP3 inflammasome senses lysosomal damage as an endogenous ‘danger’ signal [7]. We previously demonstrated impaired inflammasome activation upon inhibition of uptake by cytochalasin D pretreatment in epithelial cells [14]. Uptake of silica polymorphs, NADPH oxidase activity and ROS formation has also been linked to NLRP3 inflammasome activation in THP-1 macrophages and bone marrow-derived dendritic cells [5,31]. Shifts in the intracellular redox status resulting from an imbalance between ROS formation and antioxidant defense pathways are known to modulate innate immunity at various levels in myeloid and epithelial cells. Therefore, as expected, abrogated inflammasome signals were detected when BEAS-2B or THP-1 cells were exposed to aforementioned surface modified quartz. This was evidenced by attenuated release of IL-1β, bFGF and HMGB1, in addition to lower induction of caspase-1 activity by PVNO surface modification of DQ12 and supported by our *in vivo* findings. These data indicate that oxidant imbalances arising from particle-surface interactions may be responsible for the earlier described inflammasome activation. Also in relation to the involvement of oxidant imbalances in inflammasome activation, a key role for the thioredoxin-interacting protein (TXNIP)/TRX axis, recently coined as the “redoxisome” [42] has been evidenced [16]. Thioredoxins (TRX) reduce oxidized proteins and regulate the function of several proteins by acting as a binding partner. TRX has been for instance been described as potent ROS detoxifying protein in retinal pigment epithelial cells and has a direct effect on mitochondria by preventing oxidative stress [43]. Dostert et al. devoted a role to TRX in regulating inflammasome activation in THP1-macrophages in response to asbestos and uric acid crystals [5]. More recent work by Zhou et al. furthermore showed that lysosomal damage or increased oxidant production as a consequence of particle-cell interactions and uptake, would lead to increased dissociation of TXNIP from TRX, which was essential to inflammasome activation. Additionally, it was shown that asbestos modulates TRX/TXNIP interaction to regulate inflammasome activation [17]. We thus hypothesized that the described absence of inflammasome activation in TXNIP deficient conditions could be mimicked by increasing TRX levels. Indeed, overexpression or pretreatment with TRX1 significantly attenuated caspase-1 activation and release of IL-1β and HMGB1 in response to silica treatment. Interestingly, in previous studies we had observed significantly increased mRNA levels of TXNIP in primary normal human bronchial epithelial cells after cristobalite silica treatment [30] and confirmed cristobalite-induced increased protein levels in BEAS-2B by Western blot analysis (data not shown). These novel data suggest that the inflammasome readouts we observed are associated with caspase-1 enzyme activity in a thioredoxin dependent fashion *in vitro*. In conjunction with reported protection to induction of inflammation and airway hyperresponsiveness in a mouse model of asthma and development of emphysema and ongoing inflammation in a model of COPD by intraperitoneal injection of recombinant TRX [44,45], further investigations towards the possible therapeutic potential of TRX in silicosis and diseases characterized by inflammasome activation in general are warranted.

Taken together, these results suggest that quartz and cristobalite silica can induce inflammasome activation *in vitro* and further that exposure to the most abundant crystalline polymorph, quartz, *in vivo* is associated with inflammasome-dependent inflammatory and fibrotic remodelling of lung tissue in a surface reactivity dependent manner. Although the current study provides evidence for inflammasome activation in structural cells in addition to myeloid cells, the relative importance of inflammasome activation in these different cell types in relation to the development and progression of silicosis needs to be further investigated.

## Additional files

Additional file 1: Table S1. Histopathological scoring was performed according to schematic represented in Additional file 1: Table S1 [29]. Additional file 2: Figure S1. Determination of TRX in BEAS-2B cells that were transferred with TRX plasmid or treated with recombinant TRX protein. BEAS-2B cells were transfected or treated with TRX plasmid or TRX protein, respectively. On whole cell lysates (WCL) and concentrated supernatants (SN) the presence of TRX was measured by Western blot. GAPDH (1:20000) was used as an house keeping control. Additional file 3: Figure S2A-F. IHC images of caspase-1 and IL-1β at given time points. These supplemental figures represent immunohistochemical staining for caspase-1 on lung tissue obtained at 3 and 7 days **(2A)**, at 28 and 90 days **(2B)** as well as at 180 and 360 days **(2C)**. Representative images for IL-1β staining are indicated for different timepoints: 3 and 7 days **(2D)**, at 28 and 90 days **(2E)** as well as at 180 and 360 days **(2F)**.
