# Supplementary material for: Silica-induced NLRP3 inflammasome activation in vitro and in rat lungs
Source: Part Fibre Toxicol. 2014 Nov 19;11:58. doi: 10.1186/s12989-014-0058-0 (PMC4243278; doi:10.1186/s12989-014-0058-0)
Supplement: Additional file 1: Table S1. — Histopathological scoring was performed according to schematic represented in Additional file 1: Table S1 [29]. [file 12989_2014_58_MOESM1_ESM.doc]

**Additional file 1 Table S1**

Histopathological scoring was performed according to schematic represented in supplemental table 1 [29].

| Severity | No | Focal | multifocal | locally extensive |
| --- | --- | --- | --- | --- |
| 1 | 1.0 | 0.25 | 0.5 | 0.75 |
| 2 | 2.0 | 1.25 | 1.5 | 1.75 |
| 3 | 3.0 | 2.25 | 2.5 | 2.75 |
| 4 | 4.0 | 3.25 | 3.5 | 3.75 |
