# Supplementary figures and images for: Silica-induced NLRP3 inflammasome activation in vitro and in rat lungs
Source: Part Fibre Toxicol. 2014 Nov 19;11:58. doi: 10.1186/s12989-014-0058-0 (PMC4243278; doi:10.1186/s12989-014-0058-0)

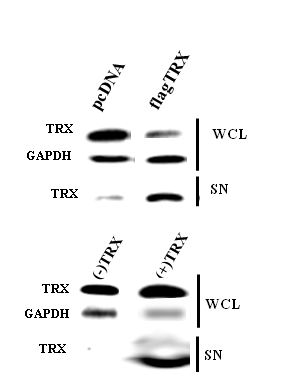

Supplement: Additional file 2: Figure S1. — Determination of TRX in BEAS-2B cells that were transferred with TRX plasmid or treated with recombinant TRX protein. BEAS-2B cells were transfected or treated with TRX plasmid or TRX protein, respectively. On whole cell lysates (WCL) and concentrated supernatants (SN) the presence of TRX was measured by Western blot. GAPDH (1:20000) was used as an house keeping control. [file 12989_2014_58_MOESM2_ESM.tiff]

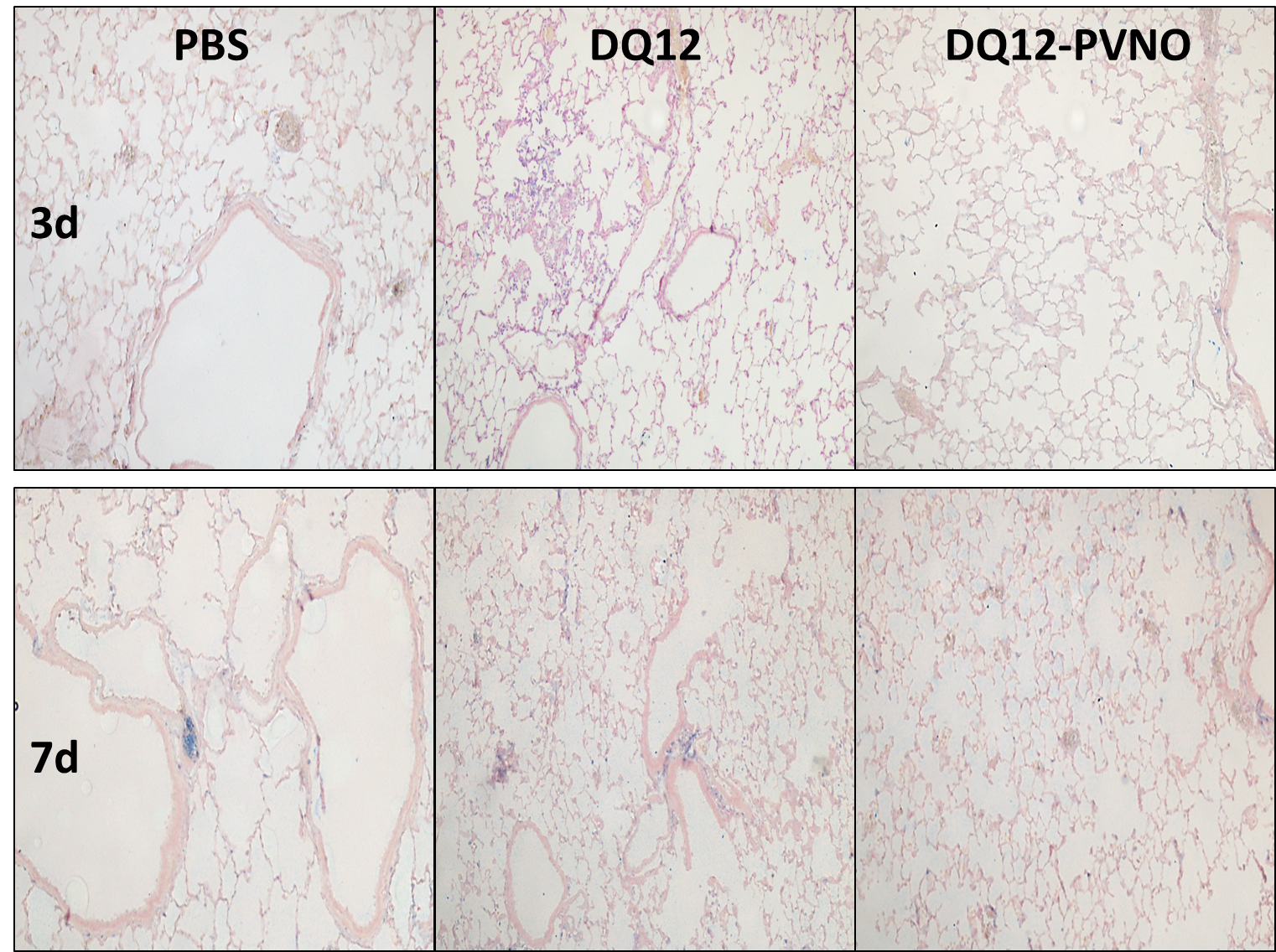

Supplement: Additional file 3: Figure S2A-F. — IHC images of caspase-1 and IL-1β at given time points. These supplemental figures represent immunohistochemical staining for caspase-1 on lung tissue obtained at 3 and 7 days (2A), at 28 and 90 days (2B) as well as at 180 and 360 days (2C). Representative images for IL-1β staining are indicated for different timepoints: 3 and 7 days (2D), at 28 and 90 days (2E) as well as at 180 and 360 days (2F). [file 12989_2014_58_MOESM3_ESM.zip › 7529516612786270_add2.tiff/7529516612786270_add2.tiff]

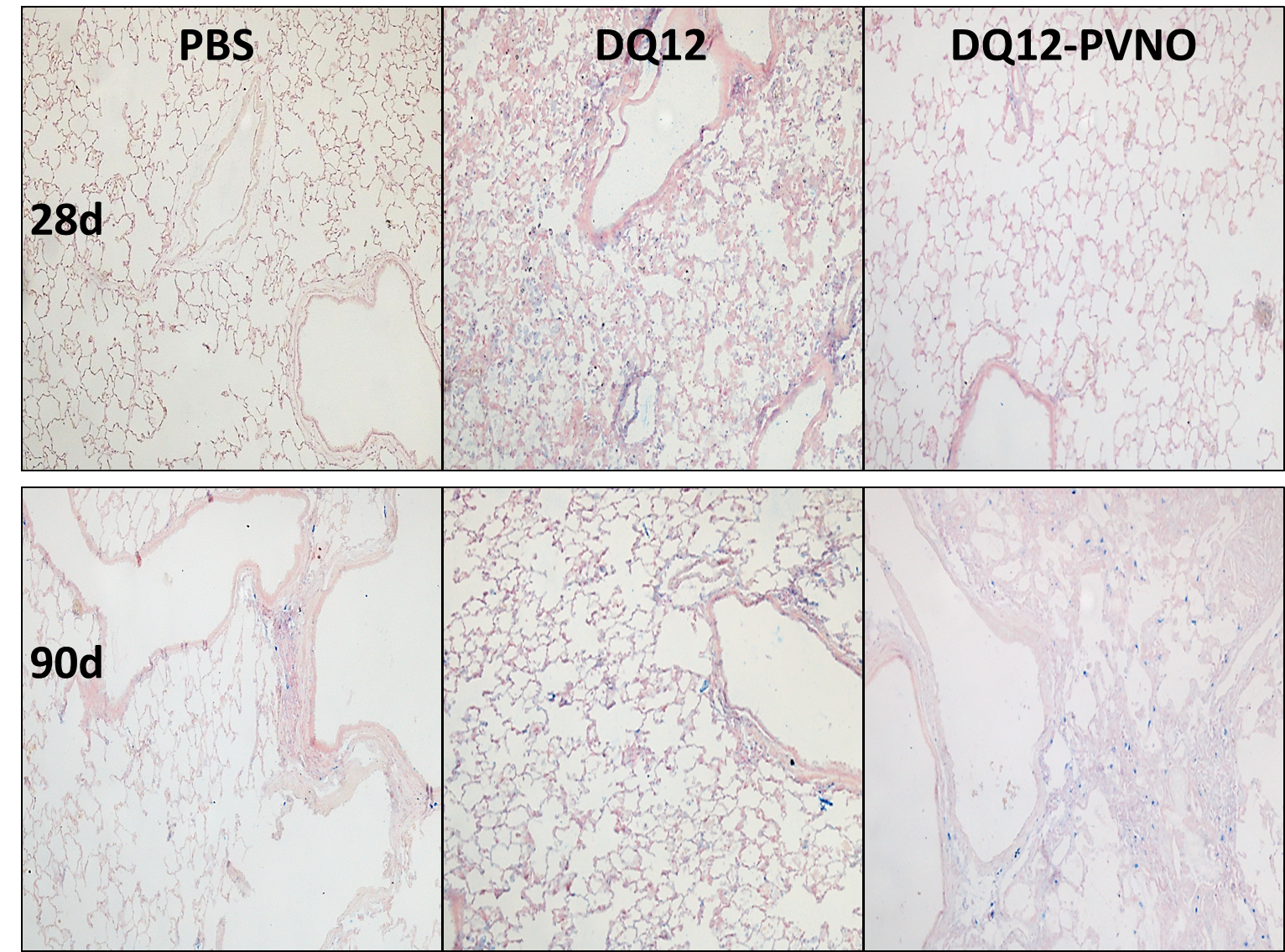

Supplement: Additional file 3: Figure S2A-F. — IHC images of caspase-1 and IL-1β at given time points. These supplemental figures represent immunohistochemical staining for caspase-1 on lung tissue obtained at 3 and 7 days (2A), at 28 and 90 days (2B) as well as at 180 and 360 days (2C). Representative images for IL-1β staining are indicated for different timepoints: 3 and 7 days (2D), at 28 and 90 days (2E) as well as at 180 and 360 days (2F). [file 12989_2014_58_MOESM3_ESM.zip › 7529516612786270_add2.tiff/7529516612786270_add3.tiff]

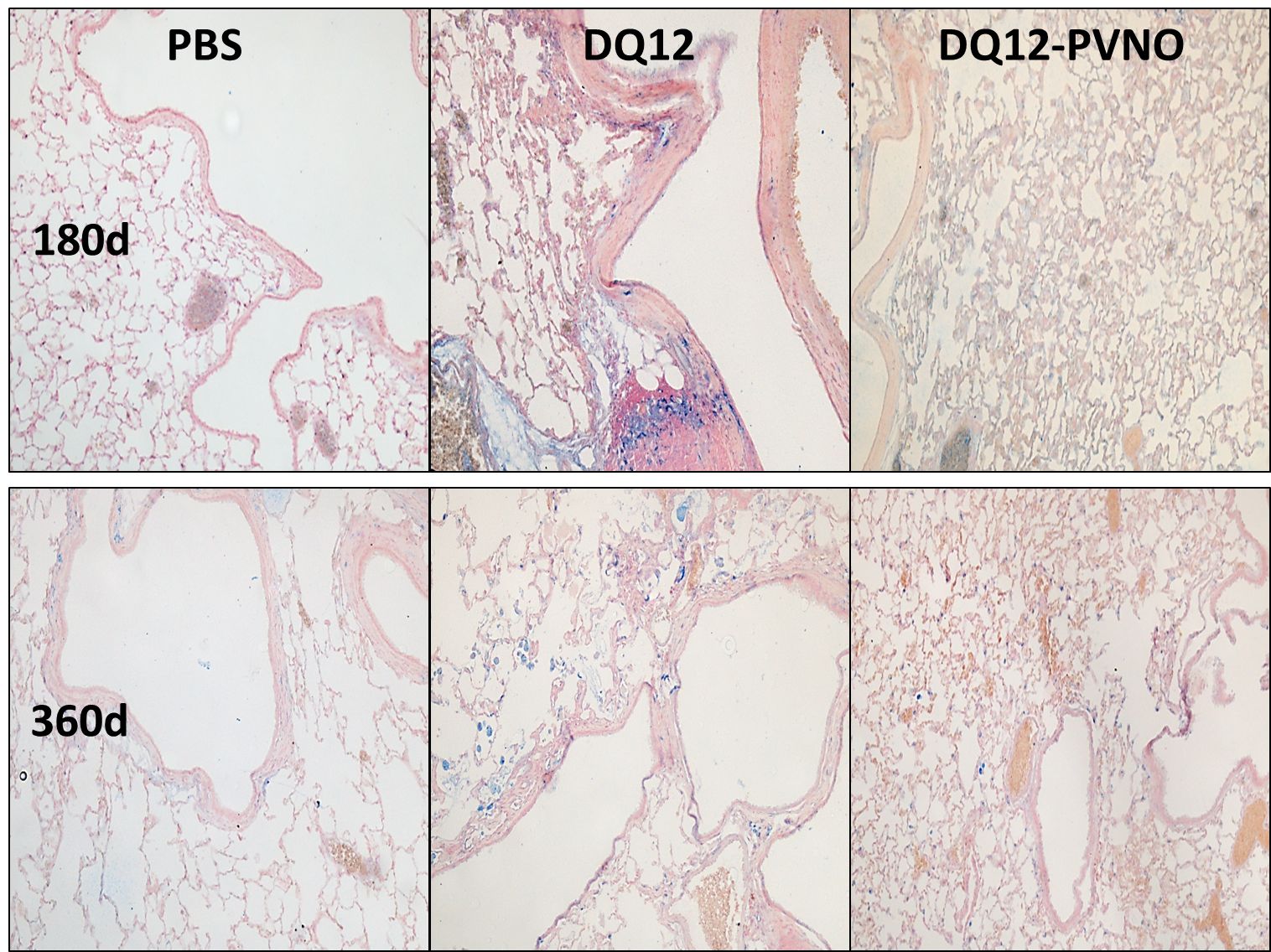

Supplement: Additional file 3: Figure S2A-F. — IHC images of caspase-1 and IL-1β at given time points. These supplemental figures represent immunohistochemical staining for caspase-1 on lung tissue obtained at 3 and 7 days (2A), at 28 and 90 days (2B) as well as at 180 and 360 days (2C). Representative images for IL-1β staining are indicated for different timepoints: 3 and 7 days (2D), at 28 and 90 days (2E) as well as at 180 and 360 days (2F). [file 12989_2014_58_MOESM3_ESM.zip › 7529516612786270_add2.tiff/7529516612786270_add4.tiff]

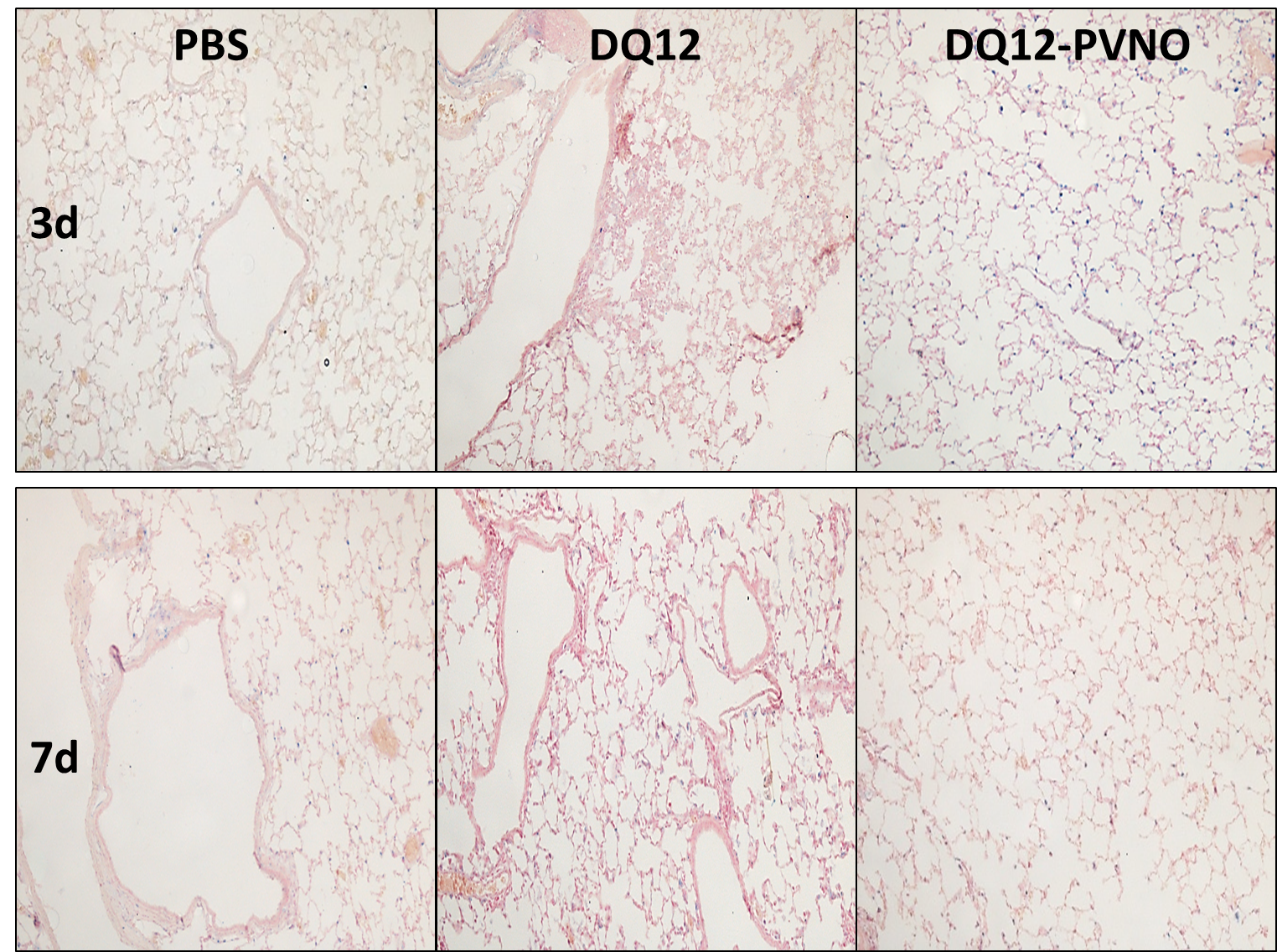

Supplement: Additional file 3: Figure S2A-F. — IHC images of caspase-1 and IL-1β at given time points. These supplemental figures represent immunohistochemical staining for caspase-1 on lung tissue obtained at 3 and 7 days (2A), at 28 and 90 days (2B) as well as at 180 and 360 days (2C). Representative images for IL-1β staining are indicated for different timepoints: 3 and 7 days (2D), at 28 and 90 days (2E) as well as at 180 and 360 days (2F). [file 12989_2014_58_MOESM3_ESM.zip › 7529516612786270_add2.tiff/7529516612786270_add5.tiff]

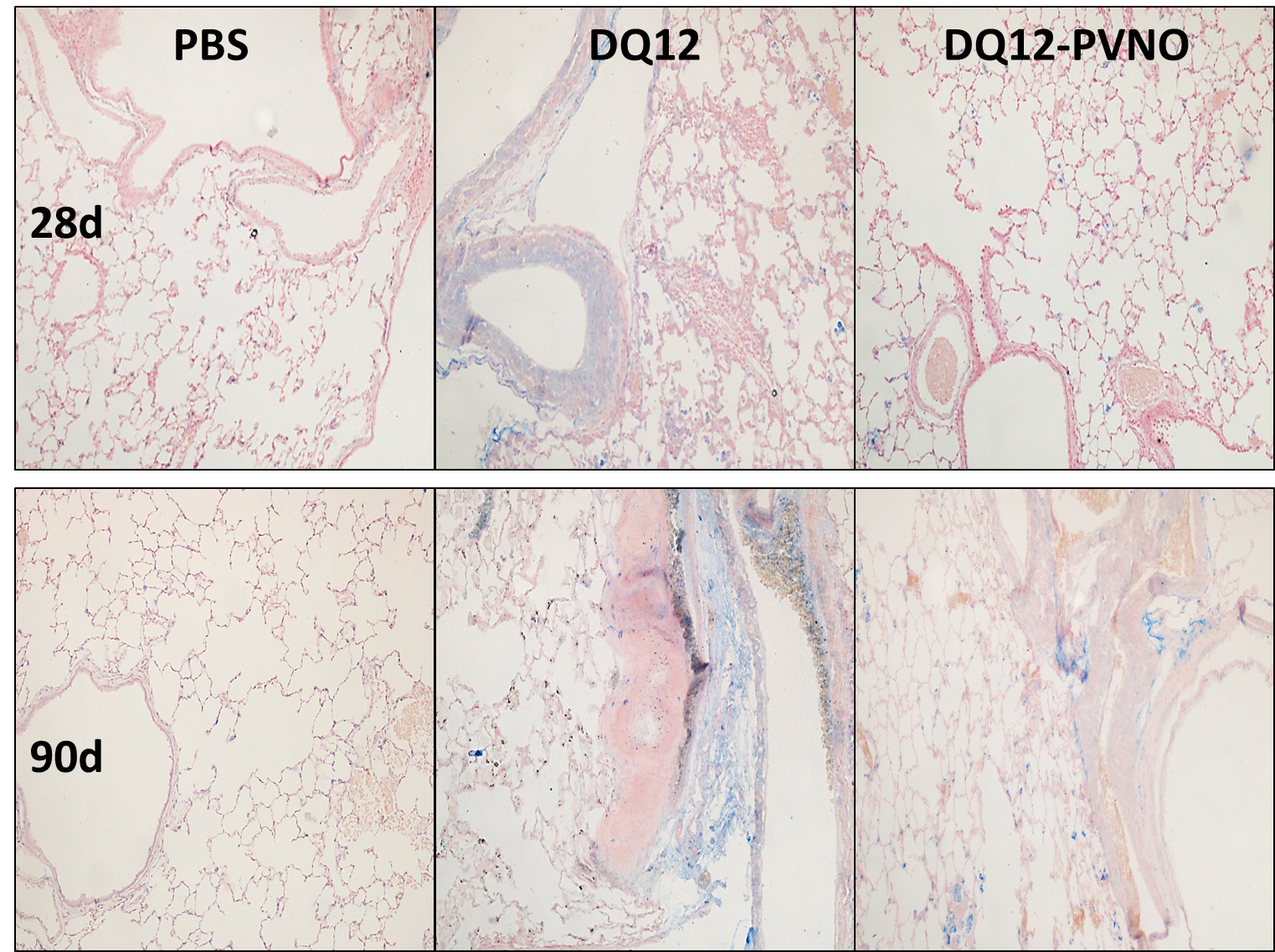

Supplement: Additional file 3: Figure S2A-F. — IHC images of caspase-1 and IL-1β at given time points. These supplemental figures represent immunohistochemical staining for caspase-1 on lung tissue obtained at 3 and 7 days (2A), at 28 and 90 days (2B) as well as at 180 and 360 days (2C). Representative images for IL-1β staining are indicated for different timepoints: 3 and 7 days (2D), at 28 and 90 days (2E) as well as at 180 and 360 days (2F). [file 12989_2014_58_MOESM3_ESM.zip › 7529516612786270_add2.tiff/7529516612786270_add6.tiff]

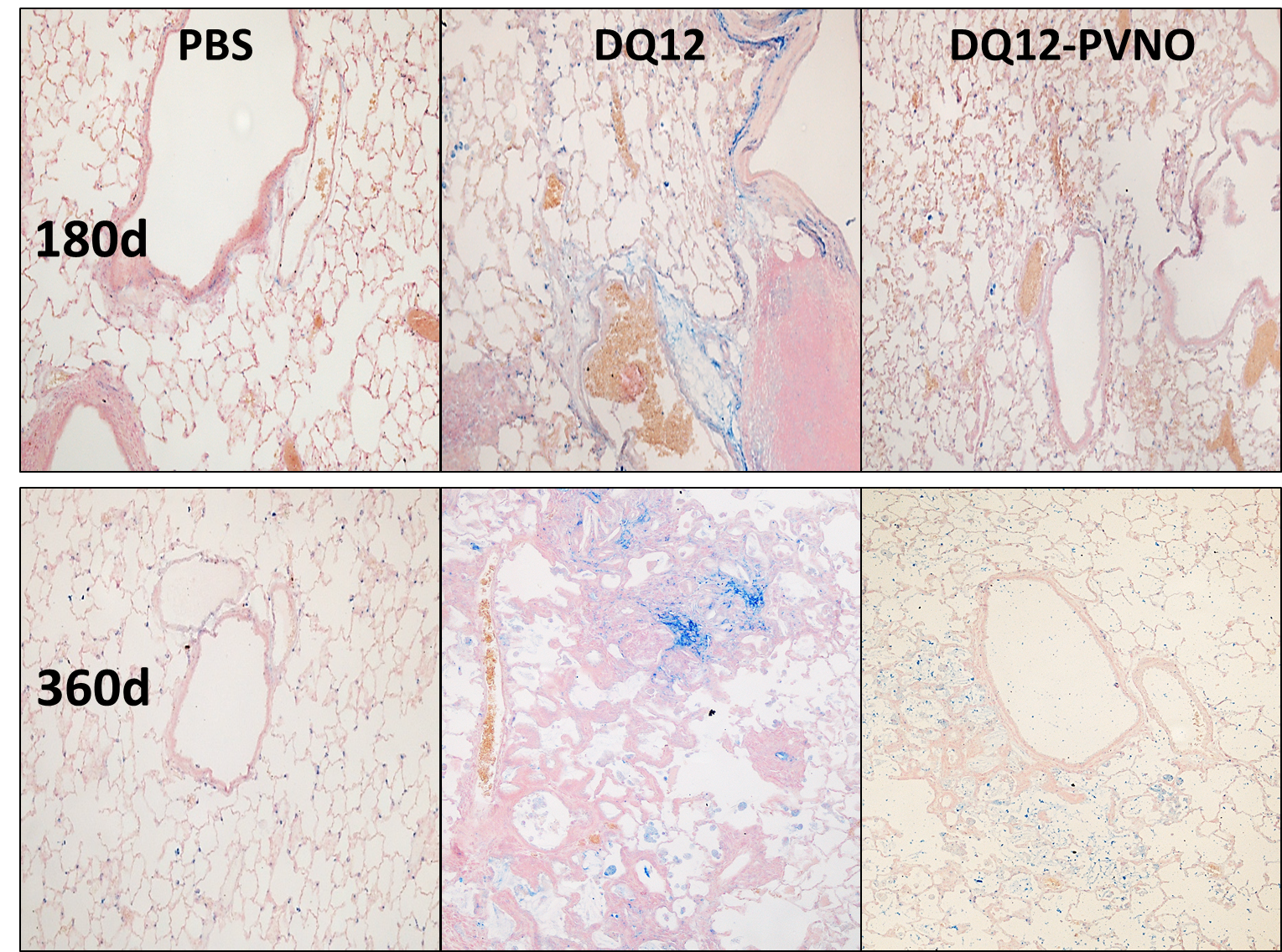

Supplement: Additional file 3: Figure S2A-F. — IHC images of caspase-1 and IL-1β at given time points. These supplemental figures represent immunohistochemical staining for caspase-1 on lung tissue obtained at 3 and 7 days (2A), at 28 and 90 days (2B) as well as at 180 and 360 days (2C). Representative images for IL-1β staining are indicated for different timepoints: 3 and 7 days (2D), at 28 and 90 days (2E) as well as at 180 and 360 days (2F). [file 12989_2014_58_MOESM3_ESM.zip › 7529516612786270_add2.tiff/7529516612786270_add7.tiff]
